# Clinical metagenomics assessments improve diagnosis and outcomes in community-acquired pneumonia

**DOI:** 10.1186/s12879-021-06039-1

**Published:** 2021-04-15

**Authors:** Fei Xie, Zhimei Duan, Weiqi Zeng, Shumei Xie, Mingzhou Xie, Han Fu, Qing Ye, Teng Xu, Lixin Xie

**Affiliations:** 1 Chinese People’s Liberation Army General Hospital, Beijing, 100039 China; 2 Vision Medicals Center for Medical Research, Guangdong, China; 3 Key Laboratory of Animal Gene Editing and Animal Cloning in Yunnan Province and College of Veterinary Medicine, Yunnan Agricultural University, Kunming, 650201 China

**Keywords:** Next-generation sequencing, Clinical metagenomics, Community-acquired pneumonia, Polymicrobial infections, Clinical study

## Abstract

**Background:**

Identifying the causes of community-acquired pneumonia (CAP) is challenging due to the disease’s complex etiology and the limitations of traditional microbiological diagnostic methods. Recent advances in next generation sequencing (NGS)-based metagenomics allow pan-pathogen detection in a single assay, and may have significant advantages over culture-based techniques.

**Results:**

We conducted a cohort study of 159 CAP patients to assess the diagnostic performance of a clinical metagenomics assay and its impact on clinical management and patient outcomes. When compared to other techniques, clinical metagenomics detected more pathogens in more CAP cases, and identified a substantial number of polymicrobial infections. Moreover, metagenomics results led to changes in or confirmation of clinical management in 35 of 59 cases; these 35 cases also had significantly improved patient outcomes.

**Conclusions:**

Clinical metagenomics could be a valuable tool for the diagnosis and treatment of CAP.

**Trial registration:**

Trial registration number with the Chinese Clinical Trial Registry: ChiCTR2100043628.

**Supplementary Information:**

The online version contains supplementary material available at 10.1186/s12879-021-06039-1.

## Background

Community-acquired pneumonia (CAP) is one of the most common and morbid conditions encountered in clinical practice [1–5]. Although some pathogens such as *Streptococcus pneumoniae* [6] are commonly detected in CAP patients, over 100 bacterial, viral, fungal, and parasitic causes of CAP have been reported [7]. Due to the limitations of culture-based testing and a lack of diagnostic tests for rare pathogens, in up to 62% of cases, the infectious cause remains unidentified despite extensive microbiological evaluation [8–10]. Failure to obtain a timely diagnosis contributes to poor clinical outcomes, increased patient anxiety, and higher costs.

Since its first reported clinical application in 2014, metagenomic next-generation sequencing (mNGS) has shown promise for the diagnosis of infectious diseases due to its ability to identify multiple pathogens by a single assay [11–13]. Recent studies have reported the validation of mNGS for pathogen detection in various specimen types and infectious diseases [14–17]. Importantly, application of clinical mNGS also led to the rapid identification of SARS-CoV-2, the causative agent for the recent COVID-19 pandemic, further highlighting its value in the diagnosis of infections [18–20]. However, most previous studies focused on its usefulness for detecting uncommon CAP pathogens [21–23], or diagnosing certain patient subgroups such as immunocompromised hosts or culture-negative cases [24–26]. Moreover, these studies mainly evaluated its diagnostic performance, and were often conducted in a relatively small cohort [21]. Prospective assessments of both the diagnostic and clinical impact of mNGS application in hospitalized CAP patients are still lacking.

In the present study, we evaluated the diagnostic performance and the impact on clinical outcome of our mNGS assay and compared the results with conventional microbiological testing in a cohort of 159 hospitalized CAP patients.

## Methods

### Cohort and study design

We recruited 159 patients admitted into the Respiratory ICU of the People’s Liberation Army General Hospital in Beijing, China from December 2018 to November 2019 with a diagnosis of CAP according to Chinese guidelines [2, 27, 28]. Patients who met the following criteria (1 + 2) and at least one of the criteria (3)–(7) were enrolled this prospective study and randomly assigned into either the control or mNGS groups with informed consents signed by patients or surrogates: (1) Admitted at our ICU and considered for pneumonia acquired outside of the hospital setting; (2) A new or progressive pulmonary infiltration with/without pleural effusion on a chest radiograph; (3) New or increased cough with or without sputum production; (4) Purulent sputum or a change in sputum characteristics; (5) Fever; (6) Signs of lung consolidation or moist rales; (7) Peripheral white blood cell (WBC) count ≥10 × 10^9^/L or ≤ 4 × 10^9^/L. Demographic characteristics of the cohort are provided in Table 1.

**Table 1:** General characteristics of the 159 patients

Characteristic	Control	mNGS	***P-value***
**Total**	100	59	NA
**Average age (**year)	74.2 (22–100)	60.4 (21–90)	< 0.05
**Sex**			
Male	73 (73%)	41 (69.5%)	0.71
Female	27 (27%)	18 (30.5%)	
**Concurrent conditions**	95 (95.0%)	52 (88.1%)	0.13
Cerebral infarction	84	4	< 0.05
Cardiovascular disease	76	24	< 0.05
Chronic lung diseases	40	13	0.02
Diabetes mellitus	25	18	0.47
Malignant solid tumor	20	7	0.27
Renal insufficiency	23	8	0.21
Immunosuppressive state	14	11	0.5
Hepatic disease	13	2	0.06
Hematologic malignancy	6	13	< 0.05

Sample collection was reviewed and approved by the Chinese People’s Liberation Army General Hospital Ethics Committee Review Board. Informed consents were signed by patients or surrogates. Patients were classified using APACHE II criteria on the first day of ICU admission [29]. The cohort was random divided into two groups. In the control group (100 cases), only standard non-NGS methods (culture or smear, acid-fast staining, T-spot and X-pert MTB/RIF for *M. tuberculosis*) were employed for pathogen detection. In the mNGS group (59 cases), samples underwent traditional clinical microbiological assays and mNGS testing in parallel.

Results of mNGS were reviewed along with other clinical evidence by the physicians, and changes in the treatment plans were made when warranted. We evaluated the impact of mNGS-based testing on clinical management and outcomes by categorizing each patient’s clinical outcome into three groups as of his/her last day in the Intensive Care Unit (ICU): Improved, Resolved, or Mortality. In the improved group, patients had resolution of abnormal vital signs, including heart rate, respiratory rate, blood pressure, oxygen saturation, and temperature; ability to eat; and normal cognition [28]. In the Resolved group, patients showed all the above signs of clinical stability and no lesions on chest CT. Patients who died during hospitalization comprised the Mortality category. In the mNGS group who had beneficial clinical outcomes, levels of alanine aminotransferase, aspartate aminotransferase, albumin, blood urea nitrogen, and creatinine were examined to evaluate the impact on patients’ liver and kidney function.

### Sample processing and sequencing

Within 2 days of enrollment, samples of blood, sputum, or bronchoalveolar lavage (BALF) were collected and transported to the laboratory following standard procedures (https://emergency.cdc.gov). Cerebrospinal fluid (*n* = 3), pleural effusion (*n* = 2), tissue (*n* = 1), and urine were also collected in a small number of cases. About 3 mL of samples were collected from the patients and stored at room temperature (for blood) or at − 80 °C (for other specimen types) prior to testing. Blood were stored in EDTA tubes, from which plasma was seperated by centrifuging at 1600 g for 10 min at 4 °C. Trypsin-liquefied sputum and BALF were centrifuged at 8000 g for 5 min. Pellets were resuspended and vortexed at 3000 rpm for 30 min in lysis buffer with the aid of glass beads to break the cell walls. DNA extraction was performed with 300 ul of specimens as described above (plasma, liquefied sputum or BALF). One hundred ng of DNA, as measured by a Qubit Fluorometer (Invitrogen, Carlsbad, CA), were then subjected to library preparation with a transposase-based method. Pooled libraries were sequenced on an Nextseq 550 sequencing system (Illumina, San Diego, CA) using a 75 bp, single-end sequencing kit.

### Bioinformatics pipeline

Raw sequencing data were first subjected to a quality control process including removal ow-quality, low-complexity, short reads (< 35 bp) and adapter trimming before further analyses. At least 10 M reads were generated for each sample. Prior to microbial classification, reads derived from the human genome were depleted by aligning to the human reference genome (hg38) using the Burrows-Wheeler Alignment (BWA) tool [30]. Microbial classification were performed by mapping the remaining sequencing reads to a reference microbial database comprising genomes of archaea, bacteria, fungi, protozoa, viruses and parasites, which was curated from the NCBI genome databases.

### Statistical analysis

Comparative analyses were conducted by Pearson’s χ2 test, Fisher’s exact test, or the McNemar test for discrete variables where appropriate. Multiple testing correction was performed using the false discovery rate (FDR) approach. Data analyses were performed using SPSS 22.0 software. Multivariate logistic regression analyses were conducted by R software, with factors of sex, age and concurrent conditions included in the model. *P-*values < 0.05 were considered significant, and all tests were 2-tailed (unless indicated otherwise).

## Results

### Sample and patient characteristics

**Fig. 1:**
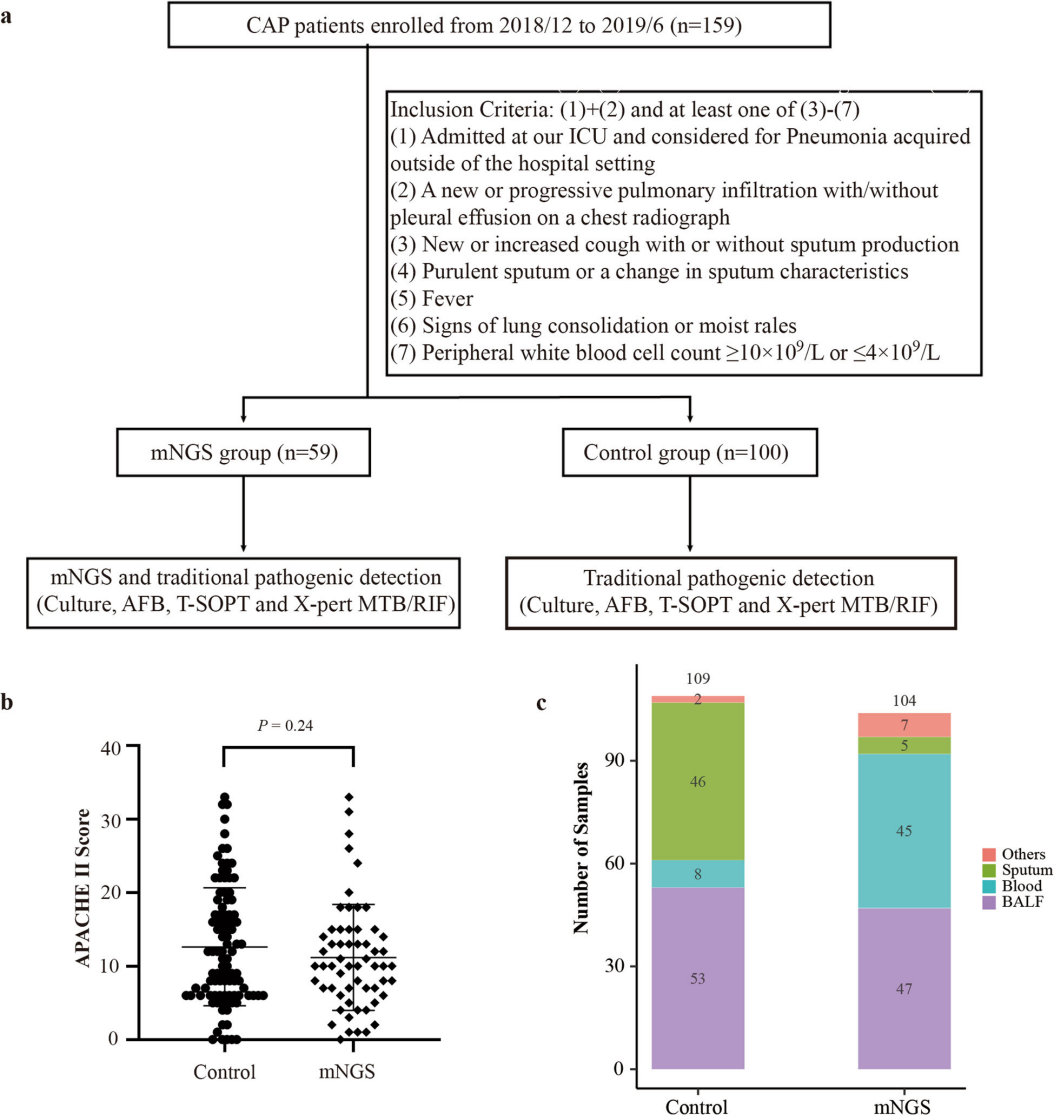
Key characteristics of patients and specimens. **a** Enrollment criteria and study design; (**b**) Comparison of APACHE II scores between the control and mNGS groups (mean of 12.6 vs 11.1, error bars represented standard deviations); (**c**) Specimens collected from the control (*n* = 109) and mNGS (*n* = 104) groups. Abbreviations: BALF, bronchoalveolar lavage fluid; CSF, cerebrospinal fluid. n.s., not significant, * indicates *P* < 0.05, ** indicates *P* < 0.01, *** indicates *P* < 0.001 by student’s *t*-test

As shown in Fig. 1a, this prospective study enrolled patients who met our inclusion criteria and randomly assigned them into either the control or mNGS groups with informed consents. The average Acute Physiologic Assessment and Chronic Health Evaluation II (APACHE II) scores were 12.6 ± 7.9 and 11.1 ± 7.0 in the control and mNGS groups, respectively (Fig. 1b). The lack of significant differences (*P = 0.24*) in APACHE scores between the two groups suggests balanced enrollment with regard to disease severity.

In the control group, a total of 109 samples were collected from 100 patients, including BALF (53), sputum (46), tissue (2), blood (8). For the mNGS group, a total of 104 samples were collected from 59 patients, including bronchoalveolar lavage fluid (BALF) (47), blood (45), sputum (5), cerebrospinal fluid (3), pleural effusion (2), tissue (1) and urine (1) (Fig. 1c). Generally, BALF and blood samples were tested in pairs in the mNGS group.

### Diagnostic performance of NGS-based Metagenomics for pathogen detection

**Fig. 2:**
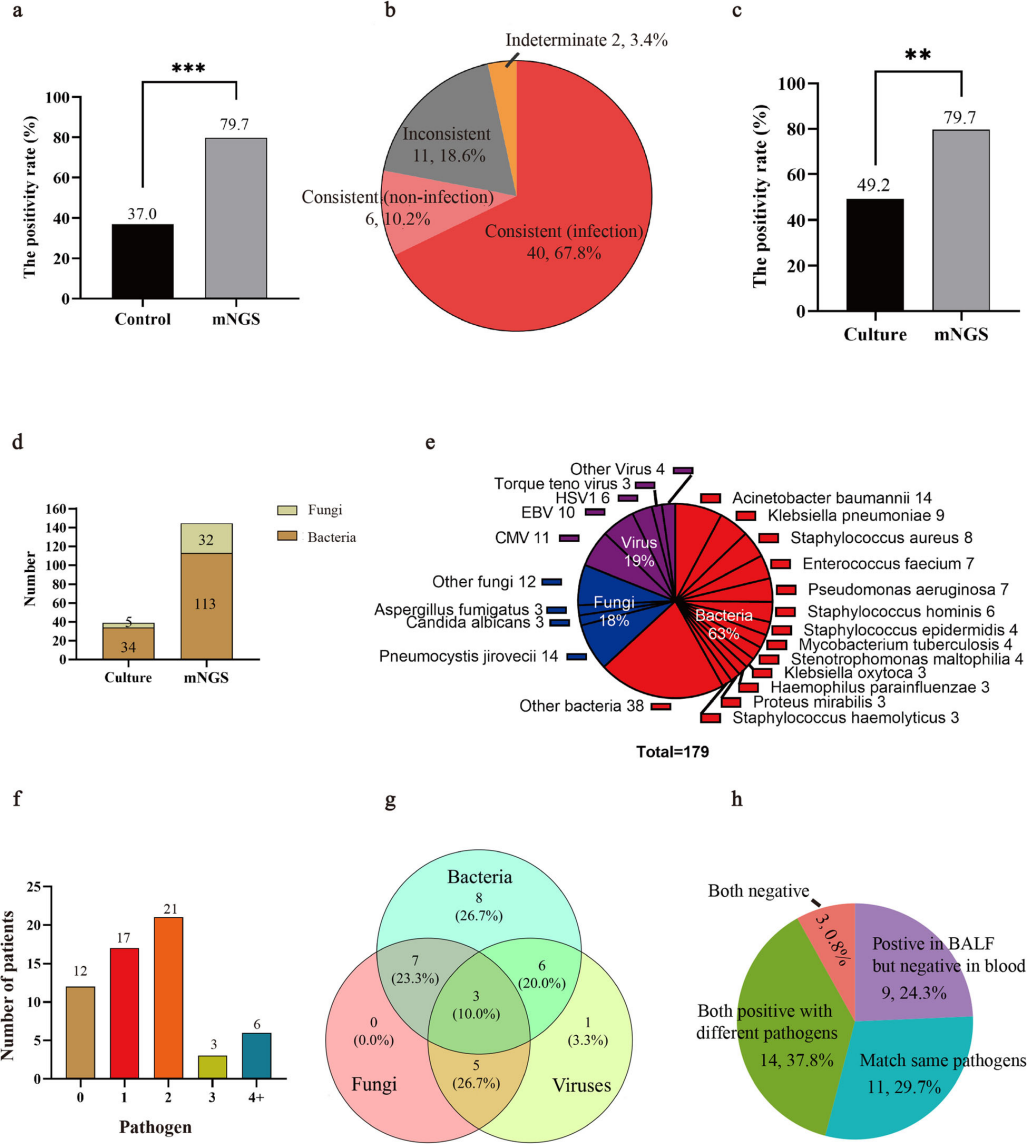
Diagnostic performance of mNGS assay for pathogen detection. **a** Rates of pathogen detection in the control and mNGS groups, summarized from testing with all collected samples; (**b**) Consistency between mNGS test results and clinical diagnosis; (**c**) Culture and mNGS detection rates for pathogens in mNGS group; (**d**) Differential spectrums of bacterial and fungal organisms identified between mNGS and culture; (**e**) Numbers of specific organisms identified by mNGS and the percentages of each microbial type; (**f**) Patients with single and multiple pathogens as identified by mNGS; (**g**) Types of pathogens shown in panel F; (**h**) Concordance in mNGS results between paired BALF and blood specimens. n.s., not significant, * indicates *P* < 0.05, ** indicates *P* < 0.01, *** indicates *P* < 0.001 by chi-square test

In this study, an NGS test was only considered positive when potential pathogen(s) were identified in the specimens. Tests that detected no microbes or only bacteria that were clinically considered commensal (for instance, *Propionibacteria spp., Veillonella spp., Rothia spp.,* and *Neisseria flavescens)* were defined as negative (Supplementary Table S1). A total of 284 pathogens were detected in the overall cohort. These included 105 pathogens (78 bacteria and 27 fungi) in the control group and 179 pathogens in the mNGS group (113 bacteria, 32 fungi and 34 viruses). In line with these findings, 79.7%(47/59) of those in the mNGS group were positive compared to 37.0% (37/100) in the controls (*P* < 0.001, Fig. 2a).

The mNGS result was considered to be in agreement with clinical diagnosis if the results confirmed either non-infection or infection with certain pathogens. For instance, two patients (a 52-year-old woman and a 51-year-old man) who had been taking immunosuppressive drugs were admitted to this study. Both had diffuse ground-glass opacity on chest CT images and but normal oxygenation index and normal levels of white blood cells, neutrophils, C-reactive protein, and interleukin-6. In both patients, *Pneumocystis jiroveci* was identified by mNGS. Based on their clinical presentations, the clinician prescribed caspofungin and compound sulfamethoxazole tablets to treat their pneumonia. Both patients recovered and were discharged. In these two cases, the results of mNGS agreed with the treatment outcome and were considered concordant with clinical diagnosis.

mNGS results agreed with clinical diagnosis in 40 (60.8%) infection cases and 6 (10.2%) non-infection cases (Fig. 2b). NGS-based assays identified significantly more pathogens (179 vs 39) in significantly more cases (29 out of 59, 49.2% vs 47 out of 59, 79.7%, *P* < 0.01) (Fig. 2c) compared to conventional techniques. A total of 34 bacterial and 5 fungal pathogens were reported by conventional testing, whereas 113 bacteria, 34 DNA viruses and 32 fungi were found by metagenomics (Fig. 2d). Discordant positive results included unculturable bacteria, viruses, and eukaryotic pathogens. The most common organisms detected by mNGS were *A. baumannii* (23.7%), *P. jirovecii* (23.7%), and cytomegalovirus (18.6%) (Fig. 2e). Our mNGS assay confirmed the diagnosis in all four cases of tuberculosis (Table 2). Multiple pathogens were identified in a substantial portion of the cases (30/59, 50.8%); in 3 cases, co-infections with bacteria, fungi, and viruses were detected (Fig. 2f, g).

**Table 2:** Diagnosis of *M. tuberculosis* using mNGS and other techniques

Pathogen detection method	Patient ID	mNGS	T-SPOT	Xpert MTB/RIF	Acid-fast staining
***M. tuberculosis***	51	154	+	–	+
	63	129	+	+	
	85	19,330		+	+
	93	2		+	+

In 37 of 59 mNGS patients, a pair of plasma and BALF samples were collected for testing. Among those, the same pathogens, including bacteria, viruses and fungi, were detected in both samples in 11 cases (29.7%). There were 3 additional cases (0.8%) in which both BALF and blood samples were negative for pathogen identification (Fig. 2h). In most of the remaining samples, BALF samples yielded better pathogen detection results than blood samples (Table 3). As indicated by the number of sequencing reads detected, the abundance of bacteria and fungi in cell-free DNA was generally lower than in the corresponding respiratory specimen (Table 3).

**Table 3:** Pathogens co-detected by mNGS in both BALF and blood samples

Patient ID	Pathogens	Reads (BALF)	Reads (Blood)
2	*Acinetobacter baumannii*	2782	68
5	*Pneumocystis jirovecii*	69	6
28	*Stenotrophomonas maltophilia*	543	10
	*Aspergillus fumigatus*	19	10
31	*Pneumocystis jirovecii*	140	1
39	*Staphylococcus aureus*	134	400
44	*Pneumocystis jirovecii*	281	288
58	*Nocardia cyriacigeorgica*	114	8
75	*Pneumocystis jirovecii*	1552	4
77	Cytomegalovirus	20,352	28,100
	Epstein-Barr virus	1610	102
	*Torque teno virus*	24	85
86	*Legionella pneumophila*	8865	2234
97	*Acinetobacter baumannii*	239,490	22

### Impact of metagenomic NGS approach on clinical management and outcome

**Fig. 3:**
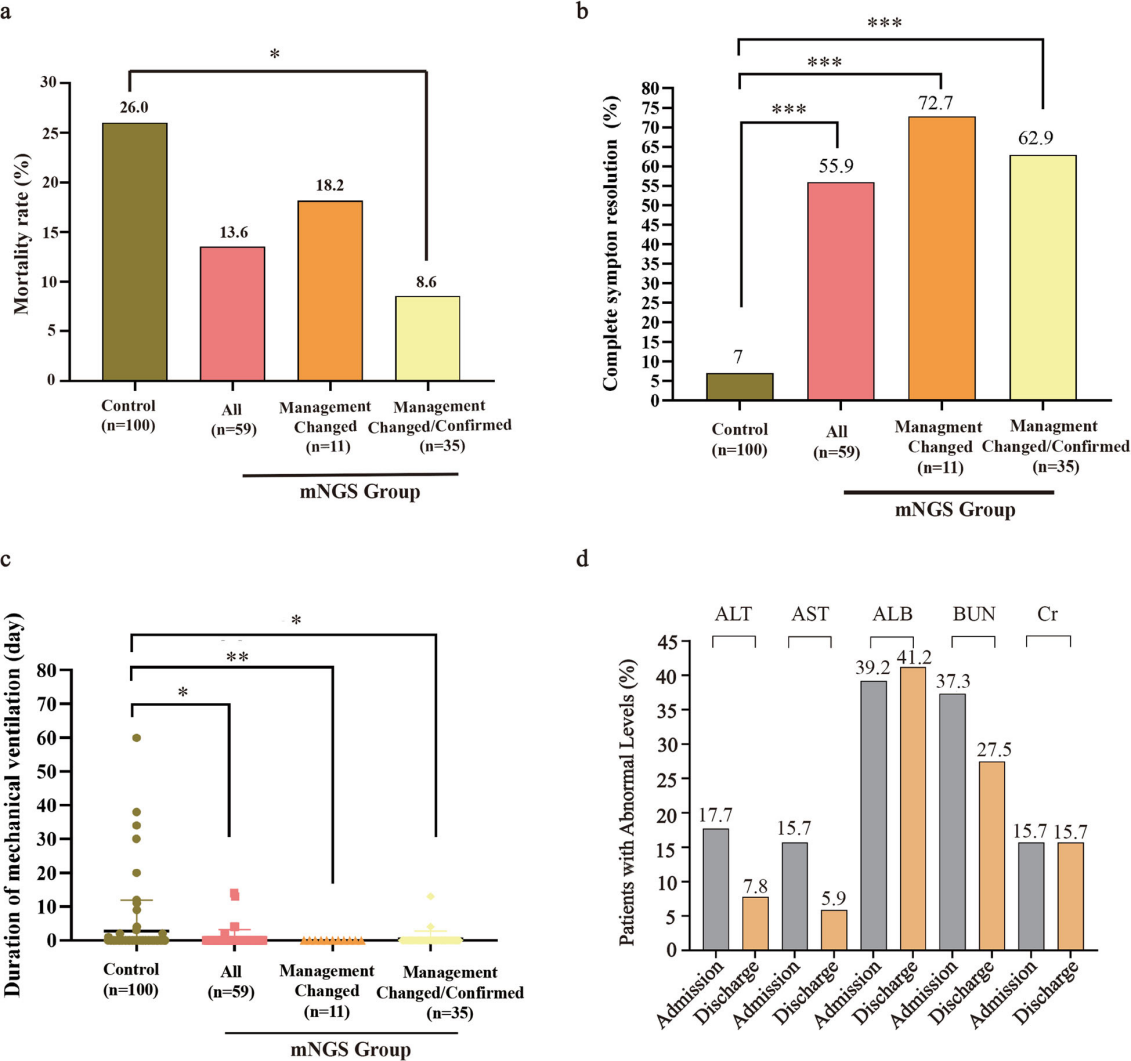
Impact on clinical outcomes by mNGS. **a** Changes in mortality rate (**b**), complete symptom resolution, and (**c**) duration of mechanical ventilation in control group (*n* = 100); mNGS group (mNGS, *n* = 59); those whose clinical management were changed based on mNGS results (Management Changed, *n* = 11); and those whose treatment was either changed (i.e. the 11 cases in the group of Management Changed) or confirmed (Management Changed/Confirmed, *n* = 35). **d** Assessment of liver and kidney function in patients with favorable clinical outcome in the mNGS group. Comparisons made between results at admission and at discharge. *, **, *** indicated *P*-values < 0.05, < 0.01 and < 0.001 respectively, by chi-square test and adjusted for multiple testing. ALT = alanine aminotransferase; AST = aspartate aminotransferase; ALB = albumin; BUN = blood urea nitrogen; Cr = creatinine

Compared to the control group, the mNGS group has a considerably lower mortality rate (13.6% vs 26.0%, or 8/59 vs 26/100; Fig. 3a) and a significantly higher rate of complete symptom resolution (55.9% vs 7.0%, or 33/59 vs 7/100, *P* < 0.001; Fig. 3b). The duration of mechanical ventilation was also significantly reduced in the mNGS group (average of 7.4 days versus 17.3 days in the control group) (*P* < 0.05, Fig. 3c). No significant differences were found in the length of stay or medical costs in the ICU (data not shown).

In the mNGS group, the results of metagenomics testing led to a) change in clinical management in 11 patients (18.6%) and b) confirmation of ongoing treatment in 24 patients (40.7%). In addition, one patient was transferred to a specialized hospital after confirmation of tuberculosis by mNGS. In the 11 cases with treatment changes in the control group, 8 showed complete resolution of symptoms (72.7% vs 7.0%, *P* < 0.001) and a decreased mortality rate (2 out of 11, 18.2% vs 26.0%, Fig. 3a, b**,** Supplementary Table S2). None of the 11 cases required mechanical ventilation.

To assess whether mNGS affected ongoing clinical management, we evaluated clinical outcomes in the 11 patients with changed treatment and the 24 patients with confirmed treatment. Consistently, compared with the control group, outcomes improved, manifested by a significantly higher rate of disease resolution (22/35, 62.9% vs 7/100, 7.0%, *P* < 0.001) and a reduced mortality rate during hospitalization (3/35 8.6%, vs 26/100, 26.0%, *P* = 0.055) (Fig. 3a, b). The duration of mechanical ventilation was also significantly shorter in 2 of the 36 cases, with an average of 8.5 days (Fig. 3c). Consistently, multivariate logistic regression analyses with factors of sex, age and concurrent conditions included also showed significant correlations between mNGS testing and improved sympton resolution (*P*<.001, control *vs.* mNGS groups; *P*<0.001, control cases *vs.* cases with treatment changed/confirmed based on mNGS results; Supplementary Table S3).

The levels of alanine aminotransferase, aspartate aminotransferase, albumin, blood urea nitrogen, and creatinine were examined in the 51 patients from the mNGS group who had beneficial clinical outcomes. As shown in Fig. 3d, fewer patients in the mNGS group had abnormal levels of these clinical indicators at discharge (compared to admission).

## Discussion

In our study, we systematically compared mNGS and standard methods in parallel for the diagnosis of CAP. mNGS analyses yielded greater pathogen detection and were associated with better clinical outcomes.

In the 14 samples that yielded a diagnosis by mNGS only (although tested by both mNGS metagenomics and culture), the causative pathogens were either not considered by treating clinicians or had tested negative by culture. These findings highlight a key advantage of the metagenomic NGS approach – it detects a broad array of potential infectious agents in a single assay. Moreover, among the 59 cases in the mNGS group, a significant portion of 31 cases were identified as polymicrobial infections by NGS, including three where co-infections of bacteria, fungi, and viruses were identified. With more clinical application of metagenomic testing, our understanding of the etiology of infectious diseases (in this case CAP) is very likely to include a more comprehensive spectrum of unculturable infectious agents and co-infections. The results of metagenomic NGS can be valuable even when they are concordant with results of conventional testing or confirm empirical treatment plans. In those cases, it provides reassurance of the diagnosis and potentially rules out non-infection cases.

Although previous studies mostly focused on the diagnostic usefulness of mNGS assays, the current study further assessed the impact of this approach on clinical management and outcomes. In our study, treatment plans were changed in 11 patients and confirmed in 24 based on the mNGS results. These patients showed significantly better outcomes than the control group. In our cohort, BALF samples were in general more likely to yield better pathogen detection compared to blood samples. This finding suggests that although testing blood samples can facilitate pathogen detection in CAP patients, they may not offer the best negative predictive value.

Currently, mNGS-based methods still have limitations. With the current technology workflow, most laboratories require a minimum of 24 h (and often 36–48 h) from sample receipt to report results [31], as the analyses consist of multiple complex steps of wet-lab processing and bioinformatics analysis. Further realization of its clinical value for infectious diseases, especially in the ICU setting, a shorter turn-around time and a less skill-demanding workflow will be very critical [32]. Furthermore, the cost of clinical mNGS (for instance, $500 per test in China) prohibits its broader application [33, 34]. However, rapid development of technology and reductions in costs of sequencing will reduce its costs and likely drive its wider adaptation [35].

With mNGS technology, a meta-transcriptomic assay could expand its capability to include RNA viruses and thus provide more clinically relevant insights into infectious diseases [36]. With the ability to analyze the transcriptomes of both the microbes and the host, distinguishing biomarkers may be identified to guide better clinical management [12, 13].

## Conclusions

Our data show that a clinical mNGS approach represents a potential step forward in the diagnosis and management of community-acquired pneumonia. This diagnostic technology may advance the identification of infectious agents, improve diagnosis and treatment, and potentially lead to favorable clinical outcome. Further research is warranted to better define, validate, and improve its clinical applications and usefulness.

## Supplementary Information

**Additional file 1.**

**Additional file 2.**

**Additional file 3.**

## Data Availability

The datasets used and/or analysed during the current study are available from the corresponding author on reasonable request.

## Abbreviations

CAP: Community-acquired pneumonia
NGS: Next generation sequencing
mNGS: Metagenomic next-generation sequencing
APACHE II: Acute physiologic assessment and chronic health evaluation II
BALF: Bronchoalveolar lavage fluid
ICU: Intensive care unit
